# Progressive resistance training and stretching following surgery for breast cancer: study protocol for a randomised controlled trial

**DOI:** 10.1186/1471-2407-6-273

**Published:** 2006-12-01

**Authors:** Sharon L Kilbreath, Kathryn M Refshauge, Jane M Beith, Leigh C Ward, Judy M Simpson, Ross D Hansen

**Affiliations:** 1 Faculty of Health Sciences, University of Sydney, Sydney, Australia; 2 Sydney Cancer Centre, Royal Prince Alfred Hospital, Sydney, Australia; 3 School of Molecular and Microbial Sciences, University of Queensland, Brisbane, Australia; 4 School of Public Health, University of Sydney, Sydney, Australia; 5 Gastrointestinal Investigation Unit, Royal North Shore Hospital, Sydney, Australia

## Abstract

**Background:**

Currently 1 in 11 women over the age of 60 in Australia are diagnosed with breast cancer. Following treatment, most breast cancer patients are left with shoulder and arm impairments which can impact significantly on quality of life and interfere substantially with activities of daily living. The primary aim of the proposed study is to determine whether upper limb impairments can be prevented by undertaking an exercise program of prolonged stretching and resistance training, commencing soon after surgery.

**Methods/design:**

We will recruit 180 women who have had surgery for early stage breast cancer to a multicenter single-blind randomized controlled trial. At 4 weeks post surgery, women will be randomly assigned to either an exercise group or a usual care (control) group. Women allocated to the exercise group will perform exercises daily, and will be supervised once a week for 8 weeks. At the end of the 8 weeks, women will be given a home-based training program to continue indefinitely. Women in the usual care group will receive the same care as is now typically provided, i.e. a visit by the physiotherapist and occupational therapist while an inpatient, and receipt of pamphlets. All subjects will be assessed at baseline, 8 weeks, and 6 months later. The primary measure is arm symptoms, derived from a breast cancer specific questionnaire (BR23). In addition, range of motion, strength, swelling, pain and quality of life will be assessed.

**Discussion:**

This study will determine whether exercise commencing soon after surgery can prevent secondary problems associated with treatment of breast cancer, and will thus provide the basis for successful rehabilitation and reduction in ongoing problems and health care use. Additionally, it will identify whether strengthening exercises reduce the incidence of arm swelling.

**Trial Registration:**

The protocol for this study is registered with the Australian Clinical Trials Registry (ACTRN012606000050550).

## Background

Upper limb arm morbidity resulting from treatment for early breast cancer is a common sequela of treatment for breast cancer [1]. In a study of 644 older women treated for breast cancer, 54% reported a decline in upper body function compared to 10% in the control group who had not undergone treatment [2]. Based on reports of upper limb morbidity [2], current practices do not adequately address or prevent the impairments that may arise from management of the tumour. While the long-term morbidity is well documented, little evidence is available to inform clinicians in the best way to minimise and prevent these upper limb problems. As upper limb morbidity is associated with reduced quality of life [3,4], particularly emotional, social and physical functioning aspects, as well as body image and lifestyle [5], it is important that effective preventive strategies be identified.

The potential benefits of progressive resistance training soon after surgery has not been investigated even though many women report shoulder weakness [4,6-8]. It is commonly believed that vigorous arm exercises will induce lymphedema [9] although evidence from our pilot study challenges this belief [10]. To date, no studies have investigated the use of resistance training early after surgery, although this may be the stage at which exercises have the greatest effect in preventing lymphedema and muscle weakness.

Stretching is advocated following breast cancer surgery; however, given the reports of reduced range and consequent shoulder stiffness [4,6-8,11], the stretching protocols do not appear to have been performed in a particularly effective way. Little detail of the actual exercises has been provided in the available randomized controlled trials [12]. In several studies, participants were seen by a physiotherapist but details of the exercises, including duration, were omitted [12]. Evidence from animal models suggests that adverse physiological adaptations can be prevented with prolonged daily stretching [13] and prolonged stretches at the shoulder have been shown to be highly effective in other patient populations at risk of developing significant loss of range [14]. Thus, prolonged stretches may decrease the loss of range and joint stiffness consequent to treatment for breast cancer.

We therefore aim to conduct a randomized controlled trial to determine whether an intensive exercise program, commencing 4 weeks after breast cancer surgery, prevents weakness, pain, swelling, and loss of range in the upper limb. We will also determine whether quality of life (as measured by arm symptoms) improves for women who undertake an intensive exercise program early after breast cancer surgery. Lastly, we will determine the effect of resistance training soon after surgery on the short-term occurrence of lymphedema.

## Methods/design

### Design

To determine the efficacy of an intensive upper limb exercise program for women soon after breast cancer surgery, we will conduct a multi-center, single-blind randomised controlled trial. Women will be recruited from 3 large hospitals with breast cancer oncology services in Sydney, Australia. Breast Cancer Nurses will inform potential participants about the study when they visit the patient after surgery. To provide sufficient time for the wound to heal, women will enter the study 4 weeks after surgery.

A record will be kept of the number of invitations to participate, the number of potential participants who volunteer to participate, and the number of screened patients who are ineligible and the reason for their ineligibility.

Ethical approval has been received from the participating hospitals as well as from The University of Sydney. All participants will provide written informed consent.

### Participants

Women will be included in the study if they: have undergone a breast cancer intervention that included surgery to the axilla; can attend for treatment and follow-up; understand English and consent to participate and be randomised to either treatment group. Women scheduled for adjunct treatments including radiotherapy, chemotherapy and breast reconstruction will be included.

Women will be excluded if they have not had surgery to the axilla or have undergone bilateral operations, present with infection at the baseline measurement or have received previous treatment for breast cancer or have metastatic disease. In addition, women will be excluded if they have sustained a fracture, undergone surgery in the upper limbs, or suffered any neurological deficit or other injury to either upper limb that may interfere with the test procedures. Lastly, women will be excluded if they undergo breast reconstruction and as a consequence, their specialist has indicated that they are not to participate.

### Randomization

An investigator who has no direct contact with the subjects will be responsible for generating the randomisation list. Randomisation will be stratified by whether women receive axillary node resection or sentinel node biopsy. The randomisation list will be used to prepare numbered opaque envelopes with the group allocation sealed inside by a person not involved in the study. Immediately after baseline assessment, women will be randomly allocated to either the usual care or exercise group. The protocol for this study is registered with the Australian Clinical Trials Registry (ACTRN012606000050550).

### Interventions

#### Usual care

Women will receive the same care as is now provided in the three participating hospitals. Women are typically admitted on the day of surgery and are discharged 2 to 7 days after surgery. While in hospital, they are seen by a physiotherapist who reviews gentle shoulder exercises which are outlined in a pamphlet. The active assisted shoulder exercises are for shoulder protraction, elevation and abduction. In addition, women are seen by an occupational therapist who discusses issues related to prevention of lymphedema. Women are not followed by either the physiotherapist or the occupational therapist after discharge from the hospital.

Women in the Usual Care group will not be given a home program of exercises, apart from those described in the pamphlet. To control for attention from the research assistant, they will meet with the research assistant fortnightly at which time their arm will be assessed with multifrequency bioimpedance analysis [15] for the development of lymphedema, but no specific advice will be given about exercise and management.

#### Exercise group

Commencing Week 4 following surgery, women will attend 8 weekly exercise sessions comprising stretching exercises and resistance training, and will commence a daily home program of similar exercises. We have used these exercises in our pilot study [10], and they are well tolerated.

Each week, there will be a supervised weekly session at which women will perform the shoulder exercises targeting the muscles and shoulder movements likely to be at risk. Stretches will consist of prolonged holds of at least 5 minutes duration. The stretches are performed in supine and are undertaken for the shoulder extensors, and for pectoralis major and minor muscles. For women unable to achieve >90 deg forward flexion, shoulder extension and abduction stretches will be performed whilst seated. For both stretches, the arm will be elevated in the appropriate direction and supported on pillows; the aim of these stretches is to provide a comfortable stretch in the region of the participant's axilla. Each of these stretches will also be held initially for 5 minutes and progressed up to 20 min. Once shoulder flexion >90 deg is achieved, the participant will undertake the stretches in supine. The muscles targeted for resistance training include the shoulder flexors, abductors, external rotators and horizontal flexors. For strength training, the resistance will be determined by the Borg Effort Scale [16] and provided by free weights. Each week, the exercises will be monitored, and progressed as required.

In addition to the weekly supervised sessions, women will perform a daily home program. The program will consist of i) a daily stretching program and ii) a strengthening program undertaken on alternate days. The stretching program will be the same as that undertaken at the weekly supervised session. For the strengthening program, the same muscles will be trained as in the weekly supervised sessions but rather than free weights, the resistance will be provided by theraband. Participants will be instructed to perform two sets of 8 – 12 repetitions for each exercise. Participants will be instructed to work at a target of 15 ('Hard') on the Borg Effort Scale [16]

At the conclusion of the program, women will be encouraged to continue the home program. A compliance diary will be provided to record the number of times each exercise was performed each day. In addition, women in both groups will be asked to record any other treatments that they have received the dose, and the effect.

### Measurement

All measurements will be undertaken by a research assistant blinded to the group to which women were allocated. Variables will be measured before randomization, at 8 weeks (the end of the intervention phase) and then at 6 months to determine the effect of the intervention on physical and psychosocial outcomes. In addition, information about the breast cancer treatment regimen, pathology report, age, weight, and previous shoulder pathology will be obtained from the patient's medical record at the baseline assessment. Occurrence of side-effects and adverse events will be evaluated at each measurement occasion.

The primary outcome measure for treatment efficacy is arm symptoms on the operated side, derived from 3 items of the Breast Module (BR23). The BR23 is a 23-item survey specific to women with breast cancer. The 3 selected items ask whether in the past week the woman has had pain in her arm or shoulder, a swollen arm or hand, and whether it was difficult to raise or move her arm sideways. For each question, there are 4 possible responses, ranging from 'not at all' to 'very much'. The raw scores for these questions are summed and then subjected to linear transformation to provide an overall score, with a maximum of 100. A high score indicates a high level of symptoms. The primary endpoint for the trial is change in primary outcome from baseline to 6 months.

Secondary outcomes include other aspects of quality of life and measures of physical impairment.

Quality of life will be measured using the European Organisation for Research and Treatment of Cancer Quality of Life Questionnaire Version 3 (QLQ-C30) and the Breast Module (BR23). These surveys are specific to cancer patients and comprise a global health status/quality of life scale, as well as functional and symptom scales. These questionnaires are reliable, valid and sensitive to change and able to distinguish patients in different disease stages and with different performance status [17-19].

Shoulder range of motion will be assessed using an inclinometer and a standardised protocol will be followed to minimise compensatory movements. Passive forward flexion, external rotation and horizontal abduction at the shoulder will be assessed in a supine position and with gravity as the standardised force. Active abduction will be assessed in the sitting position. Each arm will be measured separately. Pain at the end of the passive range of each movement will be measured using a standardised 10 cm visual analogue scale.

Maximal isometric shoulder strength will be measured objectively with a dynamometer using a standardised protocol. The arm will be positioned at 90 degrees elevation to assess forward flexion, abduction, and horizontal abduction and adduction. Movement in these planes has been selected as it has been shown to be weak following breast cancer surgery [20,21]. For each direction, women will perform 3 maximum voluntary contractions, and the highest of the three attempts will be recorded. Each arm will be measured separately.

The presence of lymphedema will be measured non-invasively using multiple frequency bio-impedance analysis. This analysis identifies early changes in tissue fluid accumulation [22] and is substantially more sensitive than total limb volume measurements in detecting changes in extracellular fluid volume. It has an extremely low (<1 %) false-positive rate for detecting lymphedema [22]. In addition, circumference measures at multiple sites on each arm will be obtained using a standardised measurement technique.

All women will be requested to record use of resources (number and type of health care contacts, medication use, adjunct treatments) during the trial period and the effect of these treatments.

The occurrence of lymphedema, regardless of treatment group allocation, is considered an 'adverse' event. If lymphedema develops, women will be referred to the Breast Cancer Nurse for referral to an appropriate specialist.

### Data analysis

#### Sample size calculations

We will recruit 180 women, 90 to each of the two treatment groups. This will give 80% power to detect as significant at the 2-sided 5% significance level a difference of 12 points (out of 100) for arm symptoms derived from the BR23, based on our pilot data standard deviation of 25. In addition, we have allowed for 20% mortality and loss to follow-up. Based on our pilot data, we will also be able to detect at least 10° difference in range of motion at the shoulder and 5 N difference in shoulder strength.

#### Statistical analysis

Treatment group will be coded to enable blinded analysis, which will be by "intention-to-treat". For the primary outcome measure, the change from baseline score for arm symptoms on the operated side derived from the BR23 will be determined. The mean change will be compared between the two groups using Student's two-sample t-test.

Our secondary analyses will examine the effect of adjusting for the stratifying factor (type of surgery) or any important prognostic factors that show a clinically important imbalance between groups. This will be done using multiple linear regression, with the baseline arm symptoms score as a covariate and the final arm symptoms score as the outcome variable.

For measures of strength, range of motion, and lymphedema, the difference between the operated and non-operated side will be determined. These differences at 6 months will be compared using a two-sample t-test or distribution-free equivalent, as appropriate. For other aspects of the quality of life score, the mean changes from baseline will be compared between the two groups using Student's two-sample t-test. As for the primary outcome, multiple regression analyses will be used for all secondary variables to adjust for covariates, including baseline differences.

## Discussion

With more than 85% of women treated for breast cancer surviving over 5 years, the long term sequelae of treatment have become increasingly important [23]. Most women are left with long-term symptoms affecting their arm and shoulder [11]. Changes to surgical management for identification of the tumour have reduced the severity of long-term sequelae but have not prevented them from occurring. In fact, of women who undergo a sentinel node biopsy, the 30% who then have an axillary node dissection in a two-step axillary operation are at greater risk of developing arm morbidity than women who only undergo axillary node clearance [5,24]. As long-term symptoms compound the physical and psychological trauma of the disease, preventing the resumption of a normal physical and emotional life [3,4], it is important to prevent their occurrence.

This randomized controlled trial was designed using features that minimise bias [25]. For example, we have included true randomisation, concealed allocation, blinded measurement and data analysis, and analysis by intention-to-treat. The nature of the treatments and outcomes precludes blinding of treatment. However, bias will be minimised by blinding subjects to the experimental hypotheses. Thus, the findings from this study will demonstrate the efficacy of an early intervention program comprising progressive resistance training and prolonged stretching.

The present proposal is to apply early, intensive exercise for prevention of side-effects from surgery. The findings from this study will enable the formulation of evidence-based data on stretching and resistance training early following breast cancer surgery.

## Competing interests

The author(s) declare that they have no competing interests.

## Authors' contributions

SK, KR and JB conceived the project. All authors contributed to the design of the trial and the procurement of funding. SK drafted the manuscript and all authors have contributed to the manuscript and have read the manuscript.

## Pre-publication history

The pre-publication history for this paper can be accessed here:


